# Intestinal Leishmaniasis in Acquired Immunodeficiency Syndrome

**Published:** 2011-05-01

**Authors:** M Molaei, M Minakari, Sh Pejhan, R Mashayekhi, A R Modaress Fatthi, M R Zali

**Affiliations:** 1 Department of Pathology, Research Center of Gastroenterology and Liver Disease, Shaheed Beheshti University of Medical Sciences, Tehran, Iran; 2 Departments of Gastroenterology and Hepatology, Isfahan University of Medical Sciences, Isfahan, Iran; 3 Department of Gastroenterology, Research Center of Gastroenterology and Liver Disease, Shaheed Beheshti University of Medical Sciences, Tehran, Iran

**Keywords:** Aids, Viscerl leishmaniasis

## Abstract

In endemic regions, visceral leishmaniasis is one of the most common opportunistic infections in HIV positive patients. Simultaneous infection with Leishmania and HIV has been reported in some countries but this is the first report of such a case in Iran. Our patient was a 27 years old man with intermittent night fever, abdominal pain, loss of appetite, vomiting, watery diarrhea and severe weight loss for 6 months. He had low socio-economic status with an imprisonment history. The patient was quite cachectic and had low grade fever. Physical exam and upper GI endoscopy revealed oropharyngeal candidiasis. Microscopic evaluation of duodenal biopsy material showed Leishmania amastigotes in macrophages of lamina propria. Leishman bodies were also observed in bone marrow aspiration specimen. Serologic tests were positive for Leishmania infantum. HIV antibody was also positive with a CD4+cell count of 80/μl. The diagnosis was acquired immunodeficiency syndrome with simultaneous visceral leishmaniasis involving intestinal mucosa.

## Introduction

Leishmaniasis is a disease of the reticuloendothelial system caused by kinetoplastid protozoa of the genus Leishmania. All species that infect humans have animal reservoirs and are transmitted by sand flies belonging to the genera Phlebotomus in the Old world and Lutzomia in the new world. The parasite assumes the amastigote form in mammalian host and the promastigote in insects.

Leishmaniasis has different clinical forms. Visceral, cutaneous and mucocutaneous forms are the best known ones. The form and severity of disease vary with the infecting species, the particular host’s immune status and prior exposure.

Visceral leishmaniasis of the Old world is caused by L. donovani or by L. infantum. The infection usually is benign and often subclinical, although some individuals, especially young children and malnourished patients, have marked involvement of the viscera, especially liver, spleen, bone marrow, and lymph nodes.[1][2][3] Visceral leishmaniasis also is an opportunistic infection in individuals with concurrent human immunodeficiency virus (HIV) disease, and the condition responds poorly to therapy in such circumstances.[4]

## Case Report

Our patient was a 27 years old man with intermittent nocturnal fever, abdominal pain, loss of appetite, vomiting, diarrhea and severe weight loss for 6 months. Patient was unemployed and had low socio-economic status. He had previous history of opioid use in forms of inhalation and digestion. He had been imprisoned 4 years ago. He denied alcohol drinking and any unsafe sexual contact or intravenous drug use.

**Fig. 1:**
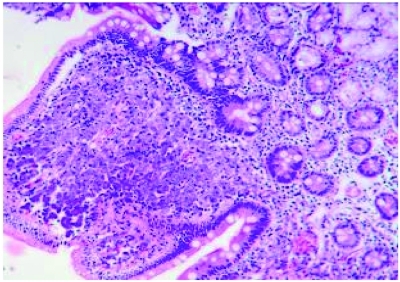
Duodenal mucosa (haematoxylin and eosine, x 400)

**Fig. 2:**
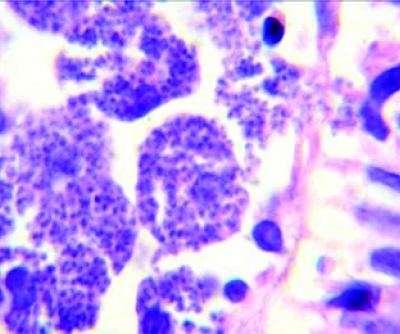
Macrophages containing Leishmania amastigotes (haematoxylin and eosine, x 1000)

**Fig. 3:**
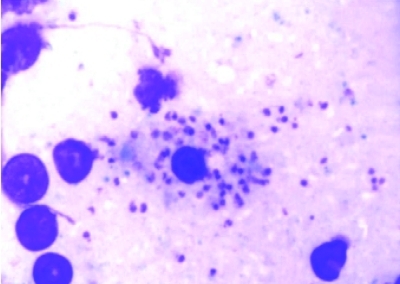
Leishmania amastigotes in bone marrow biops

**Fig. 4:**
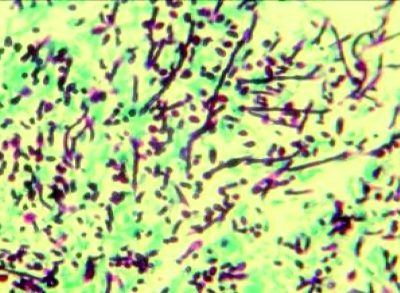
The yeast and pseudohyphae of Candida albicans (periodic acid-schiff, x400)

The patient was quite cachectic and had low grade fever. Other vital signs were normal. Physical exam revealed oropharyngeal candidiasis. He was not icteric,and no organomegaly was found. The results of his blood tests are depicted in Table 1. Upper GI endoscopy revealed severe esophagitis due to Candida with scattered whitish plaques on an erythematous basis. There were generalized nodularity and candidiasis lesions in duodenum. Microscopic evaluation of duodenal biopsy material showed partial blunting of the villi. Abundant macrophages containing intracytoplasmic microorganisms had infiltrated and expanded the lamina propria. High magnification view revealed Leishmania amastigotes with nuclei and kinetoplasts (Figure 1 and Figure 2). Leishman bodies were also observed in bone marrow aspiration specimen (Figure 3). Budding yeast cells and pseudohyphae of Candida albicans were also seen in duodenal mucosa (Figure 4).

**Table 1:** Laboratory tests of the patients

WBC( thous/mcl)	5.2
PMN (%)	82
Lymphocyte(%)	15
Hemoglobin (g/dl)	11.5
MCV (fl)	82
Platelet COUNT( thous/mcl)	240
BUN (mg/dl)	24
Creatinin (mg/dl)	0.9
AST (U/L)	24
ALT (U/L)	18
ALK-P (U/L)	224
Albumin( g/dl)	2.3
Bilirubin-T (mg/dl)	1.2
Bilirubin-D (mg/dl)	0.3
FBS (mg/dl)	75
Sodium (mEq/L)	138
Pottasium (mEq/L)	3.2
ESR (mm/hr)	32
LDH (U/L)	342
CRP (mg/L)	2+

For definite diagnosis, we checked anti-leishman antibodies in the patient’s serum. Latex aglutination test and immunofluorescent antibody were positive for Leishmania infantum.

Because of poor general condition, generalized candidiasis lesions and severe cachexia and lymphopenia, patient was suspicious for HIV infection. HIV-Ab was positive with CD4+ cell count of 80/μl, hence diagnosis of AIDS with visceral leichmaniasis was confirmed.

Patient was sent to the special center for AIDS treatment to receive the optimum care and unfortunately died 2 weeks later due to sepsis.

## Discussion

In endemic areas, visceral leishmaniasis is one of the most common opportunistic infection in HIV positive patients. Concurrent HIV infection and leishmaniasis is reported in 33 countries;[5] but this is the first report of such a case in Iran. However there have been cases of intestinal leishmaniasis without HIV infection in Iran.[6]

Most cases of HIV negative patients with visceral leishmaniasis are infants. But AIDS occurrence has changed age prevalence and clinical features of the disease. It has been suggested that Leishmania could be transmitted from human to human through needle sharing in IV drug abusers. Eighty percent of HIV positive patients with visceral leishmaniasis are male. More common high risk behavior and higher rate of IV drug abuse might be the reason of male predominance.[7] Most of HIV positive patients are affected by Leishmania infantum or donovani; but the other species such as L. braziliensis, L. major and L. tropica have been reported in their geographical areas,[8] even though leishmaniasis in HIV positive patients has the clinical features of classic Kala-azar, cutaneous and muco-cutaneous leishmaniasis in unusual sites are increasing.

Leishmaniasis in patients who are HIV positive reveals diffuse visceral involvement and is not limited to the reticuloendothelial system. These patients respond slowly to treatment and their relapse rate is high.[9] However atypical forms of leishmaniasis are seen in patients with normal immune system as well. In HIVco- infected patients, cytopenia is generally more common while organomegaly is seen less frequently.[10] In our case, visceral leishmaniasis was not associated with organomegaly, but the bone marrow was involved. Gastrointestinal involvement is relatively common in HIV positive individuals.[11] In fact, 3.2% of HIVpositive patients undergoing endoscopy for undiagnosed digestive symptoms have amastigotes.[12] Leishmania spp. can be accompanied by esophageal symptoms, epigastralgia, diarrhea, or rectal discomfort, although on occasion these symptoms are produced by other simultaneously infecting pathogens such as Cytomegalovirus or Candida spp.[13][14][15][16]

It can invade any part of the digestive tract asymptomatically; hence multiple random biopsies are indicated in spite of normal looking mucosa.[17] The polyparasitic nature of Leishmania in AIDS makes it possible to detect its amastigotes and antigen in serum. Serodiagnostic tests are not very useful in these patients. Positive response is related to the CD4 positive lymphocytes count and is seen in only 40-50% of cases. Level of anti-leishman antibody in immunocompromised patients is 50 times lower than those with normal immune system. Western blot and PCR are also accurate diagnostic tools which are truly accurate if used with ELISA and DAT. In vitro cultivation is the gold standard diagnostic method.[9] Organic pentavalent antimonials are one of the mainstays of treatment for visceral leishmaniasis; although patients co-infected with HIV have a poor response to this therapy and show a higher rate of serious side effects.[18][19]

In conclusion, visceral leishmaniasis should be considered in the differential diagnosis of gastrointestinal symptoms in HIV positive patients; especially in endemic countries and with a special attention to the impact of co-infection.
